# Testing Bottom-up Cuing Effects on Target Detection and Discrimination in Bumblebees

**DOI:** 10.1007/s10905-026-09908-2

**Published:** 2026-05-20

**Authors:** Théo Robert, Marion Callendret, Chloe Sowels, Vivek Nityananda

**Affiliations:** 1 Biosciences Institute, Newcastle University, Henry Wellcome Building, Framlington Place, Newcastle Upon Tyne, NE2 4HH UK; 2 School of Biosciences, University of Sheffield, Sheffield, S10 2TN UK; 3 Neuroscience Institute, University of Sheffield, Sheffield, S10 2TN UK

**Keywords:** Bumblebee, Insect cognition, Exogenous attention, Bottom-up attention, Insect attention

## Abstract

Attention in vertebrates helps prioritise the processing of important sensory information and filter out irrelevant signals. The capture of attention by sudden or salient stimuli is typically called bottom-up attention. Little is known about similar attentional processes in insects, although they should be advantageous for insects as well. We therefore adapted two paradigms used to investigate bottom-up attention in primates to investigate it in bumblebees: a target detection task and a target discrimination task. For both tasks, we trained bees to choose between two locations on each side of a computer screen and collect a reward below a full contrast target displayed on the screen. During detection task tests, the contrast of the target was varied, and it could be preceded by a cue flashed on the side of the target, the opposite side of the screen or not flashed at all. The discrimination task tests were similar but with a full contrast target on one side and a variable contrast distractor on the opposite side of the screen. We tested if the presence of the flash influenced the orientation and choices of the bees as well as their contrast sensitivity as has been seen in primates. We analysed bee choices using both direct observation of their behaviours at the trained locations and a trajectory analysis of high-speed videos. Our results show no effect of the prior cue, suggesting that other paradigms might prove more useful to test these processes in insects.

**Supplementary Information:**

The online version contains supplementary material available at 10.1007/s10905-026-09908-2.

## Introduction

Animals are constantly exposed to a multitude of sensory stimuli in their environment. The limited neural resources available to an animal means that these stimuli have to compete for processing and stimuli that are relevant to survival need to be prioritized. Vertebrates have therefore evolved attentional processes through which they can prioritise the treatment of the relevant sensory stimuli and enhance their perception, and filter out noise (reviewed in Anton-Erxleben and Carrasco 2013; Carrasco 2006, 2011, 2018; Carrasco and Barbot 2019; Knudsen 2007).

One form of attentional sensory enhancement is based on spatial location (Carrasco 2014, 2018). For example, humans are faster to detect a visual target at a particular location, if they are previously cued to that location (Posner 1980). Conversely, subjects take more time to detect the target if they are previously cued towards a different location in their visual field (Posner 1980). Two distinct mechanisms underlie spatial attention. The first involves a voluntary tuning of the sensory systems to increase the saliency of specific stimuli previously associated with a reward (Maunsell and Treue 2006; Scolari et al. 2014). For example, when actively looking for an object of a distinct colour in the environment, the visual cortex of primates will tune itself to prioritise the processing of the relevant colour (Saenz et al. 2002; Schoenfeld et al. 2007). Primates also show an increase in sensory sensitivity in specific spatial areas of their visual field where they direct their attention (Busse et al. 2008; Coull and Nobre 1998; Fernández et al. 2022; Ling and Carrasco 2006b; Montagna et al. 2009; Yeshurun et al. 2008). This active process, generally referred as top-down or endogenous attention, can help animals better detect relevant goals in the environment around them. Apart for primates, this type of attention has also been shown in crows and chickens (Quest et al. 2022; Sridharan et al. 2014). In the second mechanism, subjects’ attention can be captured and directed towards a region in space by salient stimuli such a flash or a loud sound through an involuntary process called bottom-up or exogenous attention (Henderson and Macquistan 1993; Liu et al. 2005; Montagna et al. 2009; Nakayama and Mackeben 1989; Theeuwes 1991; Yantis and Jonides 1984).

Bottom-up attention has been demonstrated in several vertebrate taxa. In addition to humans, other primates have also been shown to have such an attentional process (Bowman et al. 1993; Busse et al. 2008; Wang et al. 2015). Attentional processes in primates are thought to be supported by neural pathways in their neocortex (Bowling et al. 2020; Meyer et al. 2018; for a review see Behrmann et al. 2004). Similar attentional processes have, however, also been shown in birds (Quest et al. 2022; Shimp and Friedrich 1993; Sridharan et al. 2014) and possibly in fish (Gabay et al. 2013), despite the lack of a neocortex. It therefore seems likely that this is an important cognitive feature that is evolutionarily selected for and one could expect similar attentional processes to also have evolved in invertebrates.

One of the most noticeable effects of visual bottom-up attention in primates is a localised increase in contrast sensitivity (for a review, see Carrasco 2006). Cuing a subject’s attention towards a location allows them to perceive a subsequent target at that location at a lower contrast threshold, compared to when their attention is cued to another location (Barbot et al. 2011; Cameron et al. 2002; Carrasco et al. 2000; Fernández et al. 2019; Herrmann et al. 2010; Jigo and Carrasco 2020; Ling and Carrasco 2006a; Pestilli and Carrasco 2005). An increase in contrast sensitivity in response to a sudden localised change would be useful for an animal to better perceive and identify the cause of this change. It could, for example, allow the animal to quickly recognise a predator or rapidly identify potential prey.

Such adaptations would also be useful for insects. Yet, very few studies have directly investigated attentional processes in these animals (reviewed in Nityananda 2016). Weiderman and O’Carroll (2013) demonstrated that a visual neuron of a dragonfly (CSTMD1) responded selectively to one of two targets moving vertically at two different places in the visual field. More recent work showed that if one of the two locations was primed before the simultaneous presentation of the targets, the neuron was more likely to respond to the stimulus shown at the primed location (Lancer et al. 2019). Cuing effects on bottom-up attention in insects have also been shown behaviourally (Sareen et al. 2011) in the fruit fly *Drosophila melanogaster*. In this study, the authors displayed two vertical bars on a circular screen to tethered flies. When the bars moved in opposite directions on the screen, the flies had an equal probability of turning in the direction of either bar. However, if one of the stimuli flashed repeatedly before it started moving, the flies followed this bar and ignored the other one, demonstrating that the flashing bar captured the flies’ attention. The study did not, however, test if the flashing bar led to an increase of the perceived contrast of the subsequent stimuli, as has been seen in primates (Barbot et al. 2011; Cameron et al. 2002; Carrasco et al. 2000; Fernández et al. 2019; Herrmann et al. 2010; Jigo and Carrasco 2020; Ling and Carrasco 2006a; Pestilli and Carrasco 2005).

In this study, we therefore investigated the possibility that a cue could increase visual contrast sensitivity in insects. We conducted two different experiments with bumblebees, another model system for insect visual behaviour (Avarguès-Weber et al. 2011; Kelber and Somanathan 2019). In the first one, we trained bumblebees to collect a reward in a Perspex® chip placed below a full contrast target displayed on a computer screen and tested their ability to detect the target displayed at lower contrasts when it was preceded by a cue flashed at the same location as the target, a different location or not flashed at all. Our hypothesis was that if the cue led to a spatially localized increase of their contrast sensitivity, they would be able to detect the target at lower contrast when the cue was flashed at the same location compared to the other cuing conditions.

In the second experiment, bees were trained to discriminate a full contrast target from a variable contrast distractor displayed on the opposite side of the screen. During tests, a cue could be flashed on the side of the target, on the side of the distractor or not flashed in a control condition. The prediction was that when the cue was on the side of the target, it would increase its perceived contrast and enable the bees to better discriminate it from the distractor. Conversely, when the cue was flashed on the side of the distractor, if it increased its perceived contrast, we predicted that this would hinder the bees’ ability to discriminate the target from the distractor.

We analyse the bees’ choices by noting which of the target or distractor chip they probed. However, video recordings have often been used to achieve more precise analyses of bees’ behaviours such as decision making, attention, or flower approaches and strategies (De Vries et al. 2025; MaBouDi et al. 2023; Richter et al. 2023; Robert et al. 2018, 2024). Therefore, to analyse the effect of the cue in a shorter time frame, we also conducted trajectory analyses of our bees’ approach flights to the target/distractor locations from high-speed video recordings.

## Material and Methods

### Animals and Setup

**Fig. 1:**
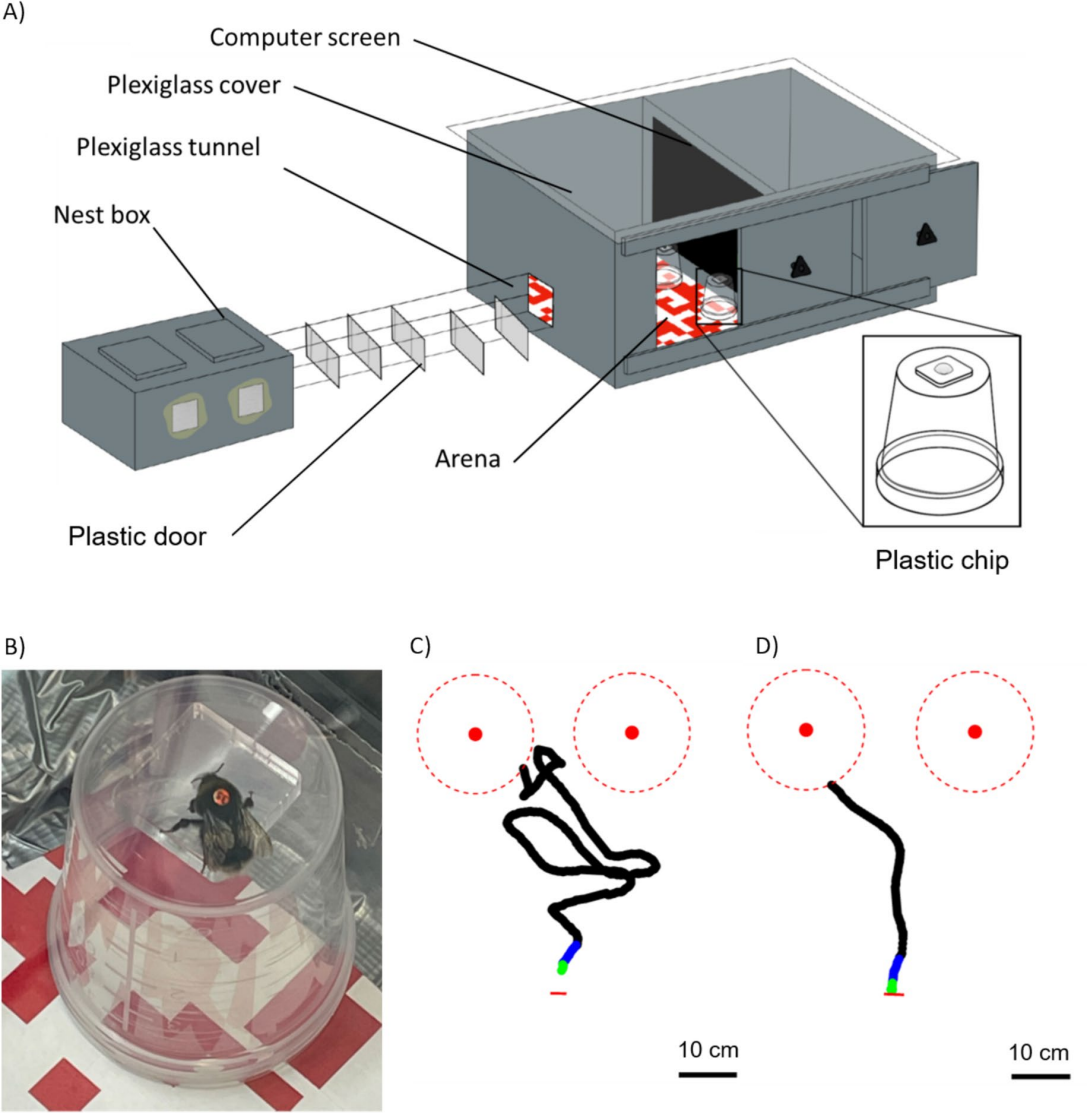
Illustration of the experimental setup and examples of approach trajectories. **A**) Schematic diagram of the experimental setup used in both the detection and the discrimination tasks. The bumblebee colony was contained in a nest box connected to the experimental arena by a plexiglass tunnel. The experimenter could use multiple plastic doors in the tunnel to select and release bees individually in the experimental arena. Experimental stimuli were displayed on a computer screen at the end of the arena and bees were trained to collect rewards from two transparent plastic chips placed on upside-down cups. **B**) Photo of an individually tagged bee drinking on one of the transparent chips on top of a transparent cup as used in our experiments. **C**) Top-down view of an example first approach trajectory recorded during a detection task test trial. The two red filled circles represent the two chips above which the target could appear. The dotted red circles around them represent the zones which we considered as approach zones (10 cm from the chip). The short red line shows the position of the tunnel entrance to the experimental arena. Trajectory sections up to 1 and 5 cm from the take-off point are marked in green and blue respectively. **D**) An example first approach trajectory recorded during a discrimination task test trial. Details as in C). Credits for the schematic diagram: Marin Nicolas

We carried out the experiments on the buff-tailed bumblebee *Bombus terrestris*. Bumblebee colonies were purchased from commercial pollinator suppliers (Koppert BV, Netherlands and Agralan Ltd, UK) and transferred to a nest box (L = 28 cm, W = 16 cm, H = 12 cm). The nest box had two chambers, one to house the brood and the rest of the colony and the other containing cat litter for the bees to dispose of their waste. The latter chamber was connected to a transparent tunnel leading to a foraging arena (L = 45 cm, W = 60 cm, H = 40 cm) (Fig. 1A). Because bees perceive UV light (Skorupski et al. 2007; Skorupski and Chittka 2010b), the arena was covered with a UV transparent plexiglass board letting through the illumination from a daylight spectrum tube (Philips, Master TL5 HE 35 W, 6500 K) fitted to a high frequency lighting system (Philips, HF-P 1 14–35 TL5 HE III, > 42kHz) with a frequency well above the 130 Hz flicker fusion threshold estimated in bumblebees (Meyer-Rochow 1981; Skorupski and Chittka 2010a). The floor of the arena was covered with a random red and white checkerboard pattern to provide the bees with optic flow (Linander et al. 2017, 2018). The arena wall facing the tunnel exit had a computer screen (Dell S2419HGF, LCD, 1080p, 144 Hz) on which we could display visual stimuli during the experiments. This screen has the advantage of a refresh rate (144 Hz) faster than the flicker fusion frequency of bees (Meyer-Rochow 1981; Skorupski and Chittka 2010a; Srinivasan and Lehrer 1985; Srinivasan and Lehrer 1984), fast response time (1 ms), and being flicker-free.

We mounted a smartphone (Huawei Nexus 6P) above the arena to record test trials at 120 fps with a 720p resolution.

The colonies had constant access to pollen placed in a little cup placed in the nest box. During evenings and weekends, feeders filled with a 20% (w/w) sugar solution were placed in the foraging arena so the bees could feed ad libitum.

### Detection Task

This experiment tested whether flashing a cue could improve or hinder target detection by bees when the cue was on the same side or the opposite side of the computer screen relative to the target. Our hypothesis and statistical analyses were preregistered at aspredicted.org (https://aspredicted.org/cnxd-vj8s.pdf).

#### Pretraining

**Fig. 2:**
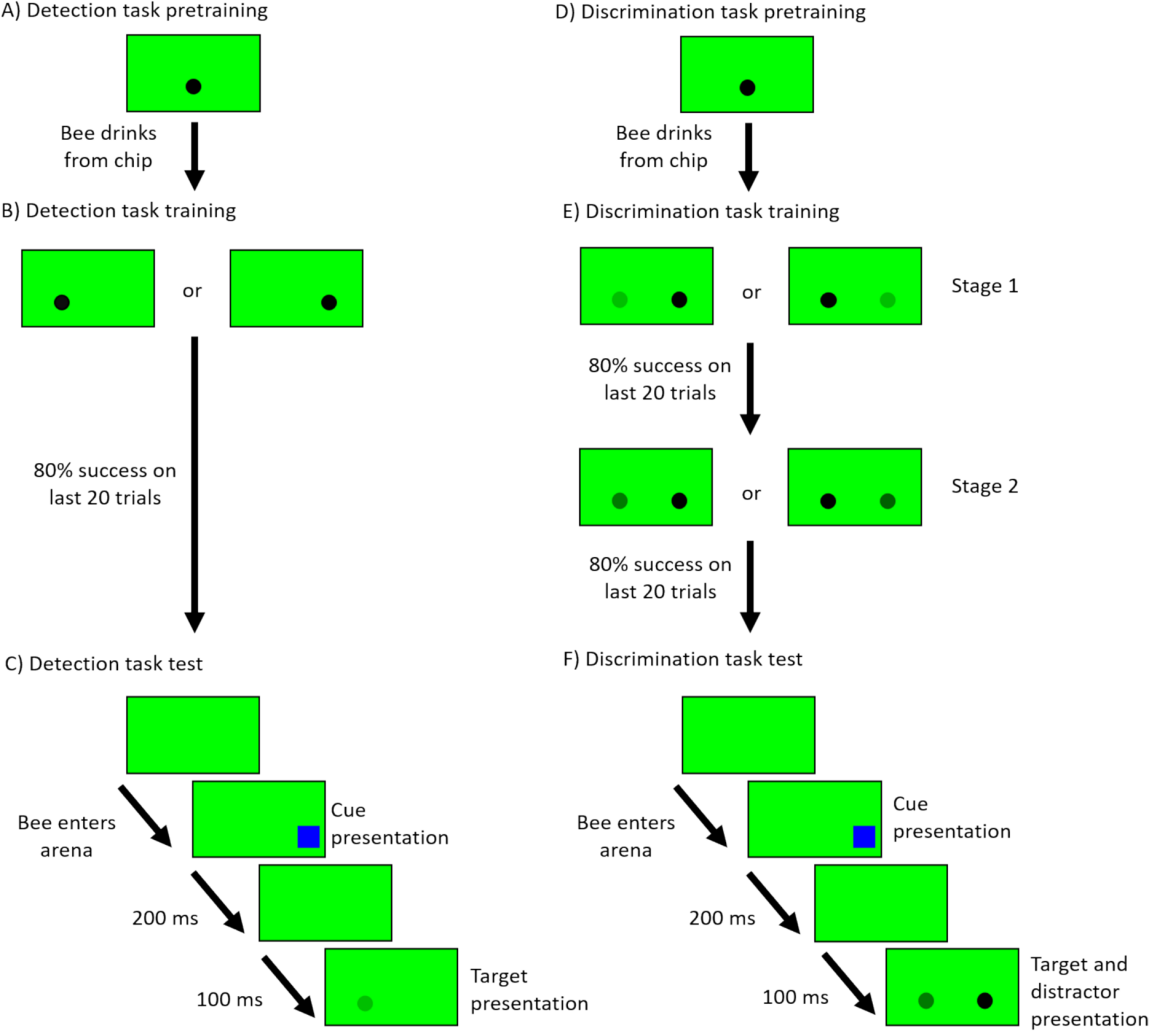
Illustration of the detection and discrimination experiments. **A**) Representation of the pretraining display for the detection task. A full contrast target was centred at the bottom of the screen and one transparent chip was placed under it. **B**) Representations of the two possible target locations during the training phase of the detection experiment. The target was always a full contrast black circle placed either on the left or the right of the computer screen. **C**) Representation of a display sequence on the computer screen during a test trial of the detection task. After the bee entered the experimental arena, a blue square cue could be flashed for 200 ms on the right side or the left side of the screen or not flashed at all. After the cue disappeared, a target was presented on either side of the screen with one of 5 possible contrasts. **D**) Representation of the pretraining display for the discrimination task. It was identical to the one for the detection task pretraining. **E**) Representations of the two training stages of the discrimination experiment. In stage 1, the target was always showed with a full contrast, either on the left or the right side of the screen, and the distractor was showed with a 0.448 contrast on the opposite side. Once the bee met the learning criterion, it moved to the second training stage during which the target was also showed with a full contrast, but the distractor could take one of 5 lower contrasts. **F**) Representation of a display sequence on the computer screen during a test trial of the discrimination task. The cuing sequence was identical to that in the detection task. During target presentation, the target was always presented at full contrast on one or the other side of the screen. On the opposite side of the screen, a distractor was displayed with one of 6 possible contrasts equal or lower to the target one

Individually marked bees were pretrained to collect a 50% (v/v) sugar solution reward from a little well at the centre of a transparent square plastic chip (L = 2.5 cm, H = 0.5 cm, Fig. 1B) placed on top of an upside-down cup (H = 7 cm, D = 6 cm) beneath the centre of the screen in the arena to avoid introducing a potential bias towards one side of the screen (Fig. 2A). This centred location was also chosen to avoid letting the bees associate the future target locations on each side of the screen with the possible stress of being manipulated by the experimenter. The aim of this pretraining was to teach bees that this type of transparent chip can contain sugar rewards. A drop of 100 µL of the sugar solution was placed in the well and the bee was placed over the chip with a transparent container. Once the bee started drinking, the container was removed, and the bee was allowed to return freely to her nest. Each bee went through the pretraining only once before moving to the training phase, to prevent bees from getting trained to the location at centre of the screen.

#### Training

After completing the pretraining, bees proceeded to a training phase (Fig. 2B). During this phase, we placed transparent chips on cups on each side of the computer screen. In each training bout, the screen was set to display a green background (RGB values: 0, 1, 0) and a full contrast black circular target (Diameter = 5.56 cm; RGB values: 0, 0, 0; 46.92 cm from the tunnel entrance) was displayed above one of the chips. The colours of the background and target were chosen because small targets are perceived by bees using their achromatic vision which is processed through their green visual channel (Giurfa et al. 1996; Giurfa and Vorobyev 1997; Hempel De Ibarra et al. 2001). Thus, our choices aimed to maximise the contrast of our target against the background. The side of the target was chosen pseudo-randomly across bouts, with a maximum of 2 consecutive bouts with the target on the same side. The chip below the target contained 100 µL of 50% Sucrose solution, while the other chip had 100 µL of distilled water. Between each trial, the chips were wiped with 70% ethanol to remove any scent marking (Pearce et al. 2017; Wilms and Eltz 2008) and then cleaned with distilled water to remove the scent of the ethanol. We deemed a trial correct when the bee first chose the chip below the target, with a choice defined as probing the contents with her antennae or her proboscis. When a bee chose the other chip, this was deemed a wrong choice, and she was allowed to correct herself and collect the reward on the correct chip before returning to her nest. Bees were allowed to proceed to the test phase once they reached 80% success on the last 20 trials (*N* = 18).

In total, 14 out of 32 bees did not complete the training and could not proceed to the test phase either because they died before completing the training, stopped coming out of their nest to forage or because they never reached the learning criterion and their training was abandoned. Among the 18 bees that completed the training, 15 managed to complete at least 1 test and were thus included in the analyses (see sections below). Due to a technical issue, the training data for 3 of these 15 bees are missing or incomplete. The remaining 12 bees with complete training data were trained for 42.083 ± 10.561 bouts (mean ± standard deviation).

#### Testing

The test phase was composed of 30 possible experimental conditions presented in a randomised order for each bee and described below, each preceded by a couple of refresher trials. The setup was identical to the training phase but both chips contained 100 µL of distilled water. During a test, the experimenter manually triggered the cuing sequence by pressing a key on the computer keyboard as soon as the bee’s head entered the arena (Fig. 2C). This sequence involved one of three conditions. In the first, a blue square cue (side 8.33 cm; centre at 49.21 cm from the tunnel entrance) was presented for 200 ms on the side of the screen where a target would later be displayed. In the second, the cue was presented on the opposite side. In the third condition, which served as a control, no cue was presented. The colour of the cue was chosen based on a previous paper demonstrating that blue stimuli disturbed shape learning in honeybees, presumably because this colour captured their attention (Morawetz et al. 2013). Its square shape was chosen to increase its difference from the target, as bees were shown to be able to discriminate shapes (Lehrer and Campan 2005; Solvi et al. 2020). The position of the cue centre on the screen was 6.64 cm from the edge of the screen and from the target location (centre to centre) to limit the risk of a forward masking effect, the reduction of the perception of a subsequent target by the display of a previous, spatially overlapping stimulus (for a review on masking effects, see Breitmeyer and Ogmen 2006). The duration of the cue was determined based on the work from Sareen and colleagues (2011) in which they captured the attention of *Drosophila* flies with a cue flashing at a 5 Hz frequency (200 ms onset to onset). For our own cue, we doubled this on-screen duration to ensure the cue would be visible by our bees. The target then appeared after a pause of 100 ms, the same off-screen duration used in Sareen and colleagues’ work (2011). Here we did not want to double this duration as we did not know how long the potential capture of the bees’ attention would last.

Each test trial presented the bees with a target with one of 5 different Michelson contrast values (Michelson contrast: 0, 0.355, 0.615, 0.826, 1). The Michelson contrast is a measure of an object contrast relative to its background and is often used in the study of contrast sensitivity in various species of bees (Chakravarthi et al. 2016, 2017, 2018; Ryan et al. 2020). To obtain our Michelson contrasts, we measured the irradiances of the targets and the background on the screen (for irradiance spectral curves and stimuli colours in the be colour space, see Fig. S3) using a spectrometer (FLAME-S-UV–VIS-ES, Ocean Optics, USA). We then calculated the Michelson contrasts with the equation $\frac{Imax - Imin}{Imax + Imin}$ with Imax the irradiance of the background and Imin the irradiance of the target (Michelson 1927). The side of presentation of the target was counterbalanced across trials and contrasts. Therefore, a complete test phase was composed of 30 trials (3 cuing conditions ×5 contrasts ×2 sides) which were presented in a randomised order for each bee. The test was considered finished when the bee landed on one of the chips and probed the well with its proboscis or antennae.

Between each test, we conducted two refresher bouts identical to the training bouts to keep the bee motivated. The side of the target during the first refresher was picked randomly and was counterbalanced for the second one. If a bee made a mistake during a refresher, it was repeated until she made a correct first choice.

Among the 18 bees who started the test phase, 15 completed at least 1 test and 8 completed all 30 tests before dying. The sample size for each combination of cuing condition and target contrast was between 20 and 26 trials.

### Discrimination Task

This experiment tested how the bee’s ability to discriminate between targets of different contrasts was affected by a cue presented on the side of the target or on the side of the distractor.

#### Pretraining

The pretraining was identical to the one of the detection task (Fig. 2D). Upon successful habituation to the feeding apparatus, bees proceeded to the training phase.

#### Training

For the training phase, the setup was the same as the detection task. The training phase was done in two stages (Fig. 2E). In the first, we presented a full contrast black circular target (diameter = 5.56 cm, Michelson contrast = 1) on one side of the screen over one chip with 100 µL of a 50% (w/w) sugar solution. On the opposite side of the screen, we showed a similar circular distractor with a 0.448 contrast above a chip filled with 100 µL of a saturated quinine solution. Quinine is an aversive substance to bees and was used to increase the cost of wrong choices. It has been shown to help bees increase their accuracy in discrimination tasks (Avarguès-Weber et al. 2010; Chittka et al. 2003; Wang et al. 2013). Our bees therefore had to learn to approach the higher contrast target regardless of which side it was displayed and to avoid the distractor. After the bees had reached 80% success on the last 20 trials in the first training stage, they proceeded to the next one. In the second training stage, the target was presented at full contrast, but the distractor had a variable contrast randomly picked without replacement from 5 possible values (Michelson contrasts: 0, 0.448, 0.680, 0.826, 0.909, for details on the calculation of these contrasts, please see the Detection task section). The list of possible contrasts was reset after the bee had experienced the 5 contrasts. Here again, a trial was marked as correct by the experimenter if the bee first chose the chip below the target by probing its well with her antennae or her proboscis. Bees had to make 80% of correct choices on their last 20 trials to be selected for the test phase. Therefore, each bee experienced each distractor contrast at least 4 times during this training phase.

In total, 34 bees started their training and 12 finished it and moved to the test phase. These 12 bees were trained for 25.750 ± 9.498 bouts on average (mean ± standard deviation) during the first training stage and 47.333 ± 17.936 bouts during the second stage.

#### Testing

The testing phase consisted of 33 possible experimental conditions, presented in a randomised order for each bee and each preceded by two refreshers trials. These 33 conditions are described below. During the tests, both chips contained 100 µL of distilled water. As in the detection task, the cue consisted of a blue square (side = 8.33 cm) 6.64 cm from the edge of the screen and from the centre of the target or distractor (Fig. 2F). Three cuing conditions were implemented: the cue could be flashed on the side of the target, on the side of the distractor or not appear at all. The duration of the cue was 200 ms with a 100 ms pause before the target and distractor were displayed. The experimenter triggered the cue as soon as the bee’s head crossed the entrance of the arena. In each test trial, the target was always at full contrast while the distractor had one of 6 contrasts (Michelson contrasts: 0, 0.448, 0.680, 0.826, 0.909, 1). Each cue-target combination could be presented on one side of the screen or the other. When the distractor was presented with a full contrast (Michelson contrast = 1), it had a contrast equal to the target stimulus. Thus, they were indistinguishable from one another. Therefore, with this distractor contrast, some conditions were visually identical. For example, the target cued condition with the target on the right side of the screen was equivalent to the distractor cued condition with the target presented on the left side of the screen. The same was true for the same conditions on the opposite sides. Thus, to reduce the total number of trials, we presented the cue only on the side of the nominal distractor in this specific contrast conditions (reducing the number of trials by 2). With the same distractor contrast, the uncued condition with the target on the right side of the screen was also identical to the one with the target on the left side. We thus removed one of these two conditions. Thus, we had 33 test trials (3 cuing conditions ×6 distractor contrasts ×2 sides – 3 trials). Here again, the test was considered complete when the bee made its final choice by landing on one of the chips and probing the well with its proboscis or antennae. The order in which theses 33 trials were presented to each bee was randomised.

Between tests, we conducted two refresher trainings, identical to the second training stage. We placed a 100 µL of a 50% (w/w) sugar reward below the target and 100 µL of quinine solution under the distractor. The target contrast was full contrast while the contrast of the distractor was one of the 5 used during the training phase. The side of presentation of each stimulus was random for the first refresher and counterbalanced for the second one. If the bee made a wrong choice, the refresher trial was repeated until she made a correct choice.

Among the 12 bees that started the test phase, 8 bees finished all tests. The sample size for each combination of cuing condition and distractor contrast varied between 10 and 20 trials.

### Video and Trajectory Analyses

Test trial recordings were processed with DeepLabCut™ (Nath et al. 2019) using a ResNet-50 network trained on a mix of 1377 video frames extracted from 68 trials from both experiments to analyse videos from the detection task. To analyse videos from the discrimination task, the network was trained on 1616 frames extracted from 78 flights from both experiments. This process allowed us to obtain the trajectories of bees. Since DeepLabCut made a certain number of errors, these trajectories were then cleaned from tracking anomalies using a custom-made code in R (version 4.2.3). All frames that were excluded as part of an anomaly were rebuilt by interpolation (The R code is available at 10.25405/data.ncl.28847825).

In order to have the most accurate take-off location for each trajectory, the videos were manually examined and the number of the frame on which the bee took off was noted. For each trajectory, if the position of the bee on the take-off frame had been interpolated, the bee coordinates were manually extracted from the video and fed back in the trajectory data.

We used the trajectories to determine which of the two chips the bee first approached in each test. This was defined as the first chip the bee approached at a distance less than 10 cm. We also measured the duration of this first approach by counting the number of video frames each bee took to complete it.

As bottom-up attention lasts for a very short time in primates (Busse et al. 2008; Hein et al. 2006; Ling and Carrasco 2006a), it was possible that the effect of the cue might be visible only at the very early stage of the flight of our bumblebees. We therefore analysed whether bees flew toward the target immediately after their take-off depending on the cuing conditions. We computed the trajectory direction of each bee relative to the target 1 cm and 5 cm away from the take-off point. To do so, we computed the angle subtended by the line joining the position of the bee when she was 1 cm (or 5 cm) from her take-off point to her take-off point and the line joining the chip below the target and the location where the bee took-off. This measures whether the direction of flight after take-off was, overall, in the direction of the target.

### Statistical Analyses

Analyses were conducted using R (version 4.2.3). We analysed the results of the detection and the discrimination experiments separately but with identical methods. We ran four analyses on our data, analysing the final choices, the first approaches, the duration of the first approaches and the direction of the early trajectories.

#### Final Choice

For both experiments, the final choice of the bee was recorded as 1 when the bee landed and probed the chip marked with the target and 0 when the bee probed the other chip. We then used Generalized Linear Mixed Models (GLMM, package lme4, Bates et al. 2015) to analyse this data with the choice as a dependent variable and a binomial family and a logit link function. The independent variables were the cuing condition (cue on the side of the target, cue on the opposite side, no cue), the contrast (the contrast of the target for the detection task, the contrast of the distractor for the discrimination task) and their interaction. We used the identity of the bee as a random effect. However, for the discrimination task data, the lack of variance in the random effect (bee identity) led to a singular fit of our model. We therefore removed the random effect and used a Generalized Linear Model (GLM) to analyse this data.

#### First Approach

The same analysis as above was conducted on the first approach data. If the first chip approached was the one below the target, this variable was marked as 1 otherwise it was marked as 0. A GLMM identical to the one used for the final choices was ran on this data. Here again, for the discrimination task data, the lack of variance in the random effect led to a singular fit of our model. We therefore removed the random effect and ran a GLM on the data.

#### First Approach Duration

We also analysed the duration of this first approach with GLMMs using the glmmTMB function from the glmmTMB package (Brooks et al. 2017; McGillycuddy et al. 2025). The dependent variable was the time in seconds between the take-off and the point at which the bee was less than 10 cm away from one of the chips for the first time. In one model, we tested the effect of the contrast of the target (for the detection task) or of the distractor (for the discrimination task), the cuing condition and their interaction. While in a second model, the independent variable was the contrast, the chip chosen by the bee (correct or incorrect chip) and their interactions. These models were fitted with a Gamma family and a log link function. To improve the fit of our models used to analyse the approach duration in the detection task, the dependent variable had to be transformed by applying a log10 function to it. Finally, for both experiments, the dispersion of the data had to be modelled by providing a dispersion formula in the glmmTMB function. This dispersion was modelled based on the distractor contrast, the cuing condition and whether the chosen chip was correct or not (Dispersion formula = Contrast*Cuing condition*Chosen chip).

#### Early Flight Direction

We tested for the effect of the cue on the initial flight direction of the bees by analysing the bee trajectory direction relative to the target at 1 and 5 cm from their take-off point. To do so, we separated trials with the target on the right and left sides of the screen. Then, for each side group, we pooled all trials for each cuing condition and used the Rayleigh test from the “circular” R package (Agostinelli and Lund 2024) to test whether each group’s flight direction was significantly oriented towards 0 (perfectly flying towards the target).

In addition, we used the Watson-Wheeler test from the same package to test whether bee early flight direction differed between cuing conditions in each of the side groups.

### Sample Sizes

We applied various exclusion criteria on our test flights for the different analyses we conducted (details provided in supplementary information). The final sample sizes for all tests are provided in the statistical tables.

## Results

### Detection Task

#### Final Choice Analysis

**Fig. 3:**
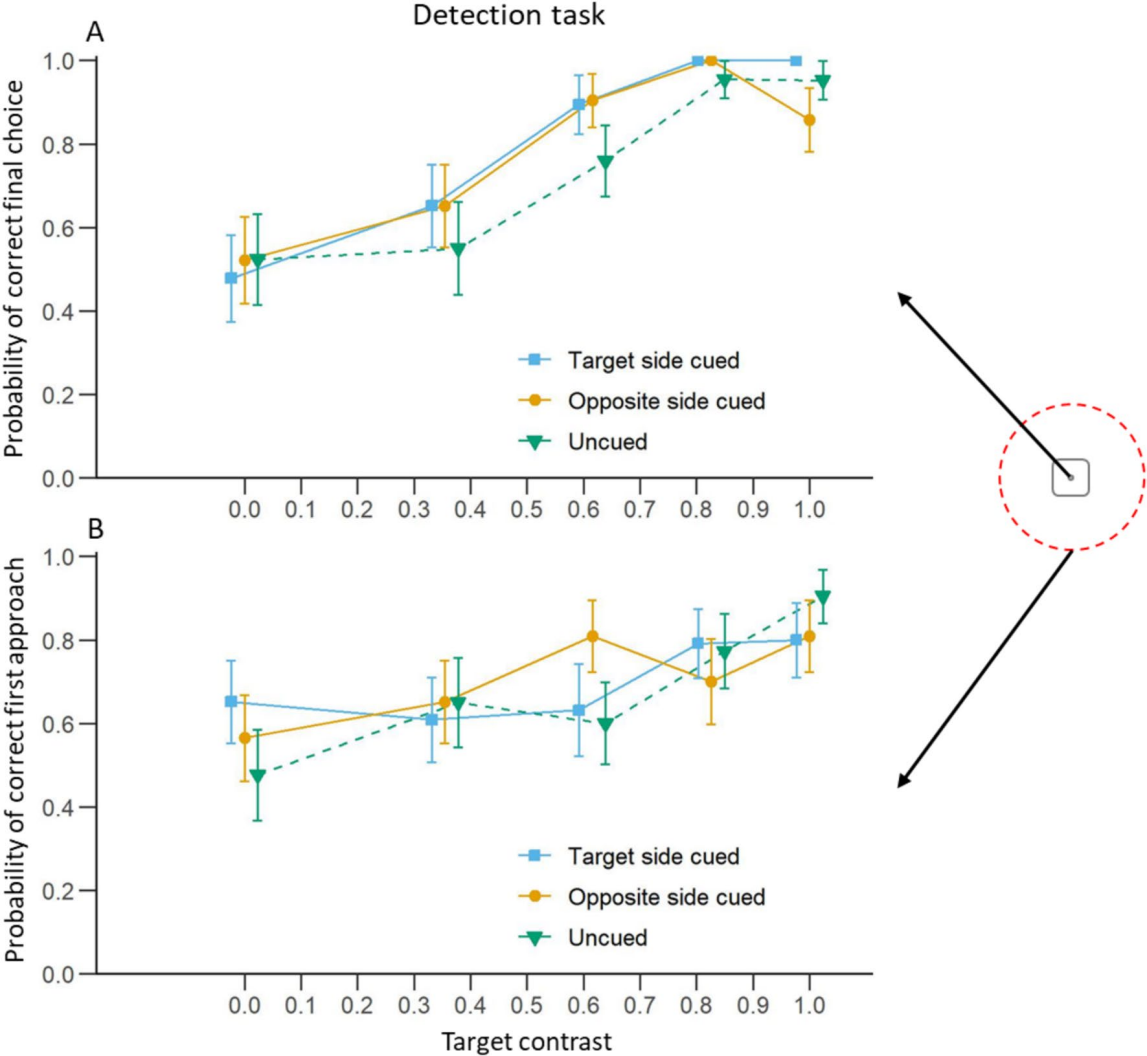
Detection task: Effect of target contrast and cuing condition on the probability of choosing the target side. **A**) Mean (± S.E.) proportion of trials in which bees chose target chips as a function of the Michelson contrast of the target. **B**) Mean (± S.E.) proportion of trials in which the bee first approached the target chip at a distance less than 10 cm. Blue curves represent trials where the cue was presented on the same side as the target. Yellow curves represent trials where the cue was presented on the opposite side to the target. Green curves represent trials without a cue. The schematic on the right shows a top-down view of a transparent chip. A final choice was when the bee probed the well at the centre of the chip with her antennae or her proboscis. The red dashed circle represents the 10 cm radius from the centre of the chip. A first approach was when the bee crossed the 10 cm radius around one of the two chips for the first time

The probability of a correct final choice during tests increased with an increase in the target contrast in all three cuing conditions (Fig. 3A; Table 1A). However, compared to the uncued condition, the display of the cue on the target side or on the opposite side of the screen did not affect the chances of correct choices. There was also no significant difference between the probability of a correct final choice between the two cued conditions.

**Table 1:** Results from the Generalised Linear Mixed Models (GLMM) used to analyse the final choice and first approach data for the detection task

A) Final Choice	GLMM	*N* = 326		
Tested effect	Estimate	Std error	Z	p
Target contrast (for Uncued)	2.901	0.740	3.920	< 0.001
Target contrast (for Target side cued)	4.457	0.977	4.561	< 0.001
Target contrast (for Opposite side cued)	2.728	0.757	3.605	< 0.001
Monitor side cued (Target side cued VS Uncued)	−0.096	0.561	−0.171	0.864
Monitor side cued (Opposite side cued VS Uncued)	0.335	0.550	0.608	0.543
Monitor side cued (Opposite side cued VS Target side cued)	0.430	0.550	0.782	0.434
Interaction Target contrast : Monitor side cued (Target side cued VS Uncued)	1.556	1.218	1.278	0.201
Interaction Target contrast : Monitor side cued (Opposite side cued VS Uncued)	−0.172	1.054	−0.164	0.870
Interaction Target contrast : Monitor side cued (Opposite side cued VS Target side cued)	−1.729	1.232	−1.403	0.161
B) First Approach	GLMM	*N* = 264		
Tested effect	Estimate	Std error	Z	p
Target contrast (for Uncued)	1.829	0.627	2.917	0.004
Target contrast (for Target side cued)	0.856	0.592	1.146	0.148
Target contrast (for Opposite side cued)	1.099	0.605	1.816	0.069
Monitor side cued (Target side cued VS Uncued)	0.611	0.530	1.154	0.248
Monitor side cued (Opposite side cued VS Uncued)	0.532	0.521	1.004	0.315
Monitor side cued (Opposite side cued VS Target side cued)	−0.080	0.520	−0.154	0.878
Interaction Target contrast : Monitor side cued (Target side cued VS Uncued)	−0.973	0.861	−1.130	0.258
Interaction Target contrast : Monitor side cued (Opposite side cued VS Uncued)	−0.730	0.870	−0.839	0.401
Interaction Target contrast : Monitor side cued (Opposite side cued VS Target side cued)	0.243	0.846	0.287	0.774
C) First Approach Duration (depending on cueing conditions)	GLMM	*N* = 264		
Tested effect	Estimate	Std error	Z	p
Target contrast (for Uncued)	−0.182	0.191	−0.950	0.342
Target contrast (for Target side cued)	−0.470	0.151	−3.119	0.002
Target contrast (for Opposite side cued)	−0.387	0.124	−3.126	0.002
Monitor side cued (Target side cued VS Uncued)	0.136	0.170	0.800	0.424
Monitor side cued (Opposite side cued VS Uncued)	0.013	0.156	0.080	0.936
Monitor side cued (Opposite side cued VS Target side cued)	−0.124	0.127	−0.972	0.331
Interaction Target contrast : Monitor side cued (Target side cued VS Uncued)	−0.289	0.240	−1.202	0.229
Interaction Target contrast : Monitor side cued (Opposite side cued VS Uncued)	−0.206	0.229	−0.898	0.369
Interaction Target contrast : Monitor side cued (Opposite side cued VS Target side cued)	0.083	0.197	0.423	0.672
D) First Approach Duration (depending on first approach correctness)	GLMM	*N* = 264		
Tested effect	Estimate	Std error	Z	p
Target contrast (for Correct first approaches)	−0.477	0.100	−4.781	< 0.001
Target contrast (for Incorrect first approaches)	0.048	0.139	0.344	0.731
First approach correctness (Correct first approaches VS Incorrect first approaches)	−0.182	0.104	−1.745	0.081
Interaction Target Contrast : First approach correctness	0.525	0.172	3.058	0.002

The table presents the number of trials (N) used in each GLMM. It also shows the estimates, standard errors, Wald test results and *p*-values for each tested factor in each GLMM. The GLMMs tested the effect of the target contrast, the cueing condition, and their interaction on the probability of correct first choice (A), on the probability of correct first approach (B) and on the duration of the first approach (C). A last model tested the effect of whether the first approach was to the target (correct first approach) or the other side of the monitor (incorrect first approach) on the duration of this first approach

#### First Approach Analysis

As in the final choice analysis, an increase in target contrast increased the probability that the bees approached the correct chip in the uncued condition (Fig. 3B; Table 1B). Although a similar trend was observed in the two cued conditions, the effect of the target contrast was not significant. Despite this, when compared to the uncued condition, we did not find a significant effect of the cue (main effect or interaction with the target contrast) flashed on the side of the target or on the opposite side on the probability of correct first approach. In addition, a cued displayed on the side of the target did not improve the probability of a correct first approach compared to a cued displayed on the opposite side of the screen.

#### First Approach Duration Analysis

**Fig. 4:**
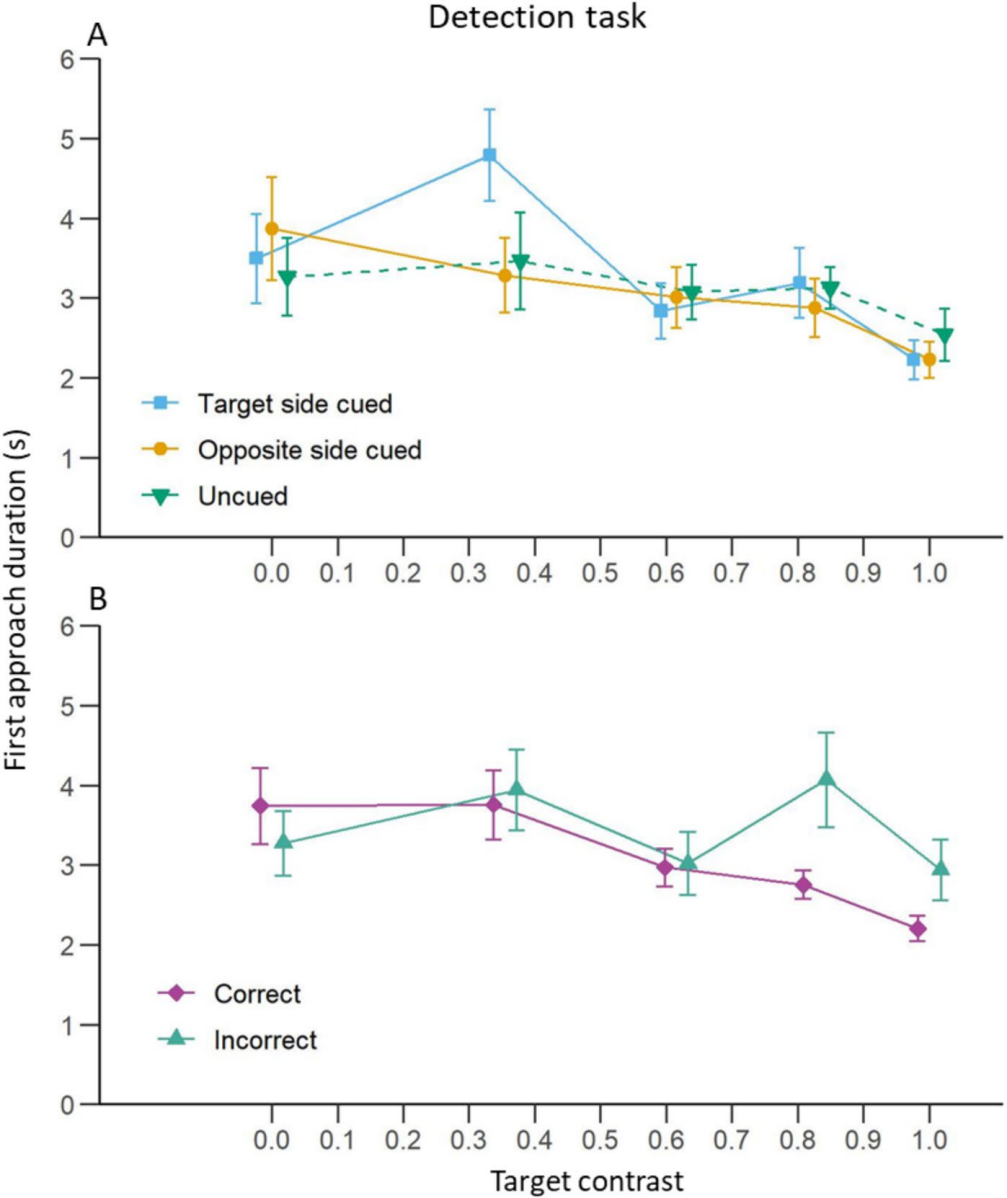
Detection task: Effect of target contrast on bee first approach duration.** A**) Mean (± S.E.) time taken by the bees to first approach either chip at less than 10 cm for each cuing condition. The blue curve represents trials where the cue was presented on the same side as the target. The yellow curve represents trials where the cue was presented on the opposite side to the target. The green curve represents trials without a cue. **B**) Mean (± S.E.) time taken by the bees to first approach the chip at less than 10 cm for trials where bees made a correct (purple) or incorrect (turquoise) first approach

We first ran a model investigating the effect of contrast and cuing condition. We found a non-significant trend that the first approach duration decreased with the increase of the target contrast when the cue was not displayed (Fig. 4A; Table 1C). However, the same effect was significant in both conditions when the cue appeared. There was no significant effect of the cue when comparing both cued conditions to the uncued one. There was also no interaction between the cueing condition and the target contrast. Here again, this absence of significant interaction tends to support the fact that overall, the cue did not have an effect on the first approach duration but the more the target was visible, the faster the bees made their choice. There was also no difference in first approach duration between the two cued conditions themselves.

To investigate the effects of choice accuracy on the first approach duration, we ran a second model with contrast and choice accuracy (correct or incorrect) as independent variables. We found a significant interaction between the two variables: bees made faster first approaches with the increase of the target contrast when they approached the correct chip (Fig. 4B; Table 1D) but not when they approached the wrong one. This interaction indicated that although the first approach duration was similar between chips when the target was invisible, i.e. with a contrast = 0, the effect of the target contrast differed depending on whether the animals were approaching the target chip or the other chip. This may indicate that the bees were more hesitant when approaching a chip when the target was not discernible.

#### Early Flight Direction

**Fig. 5:**
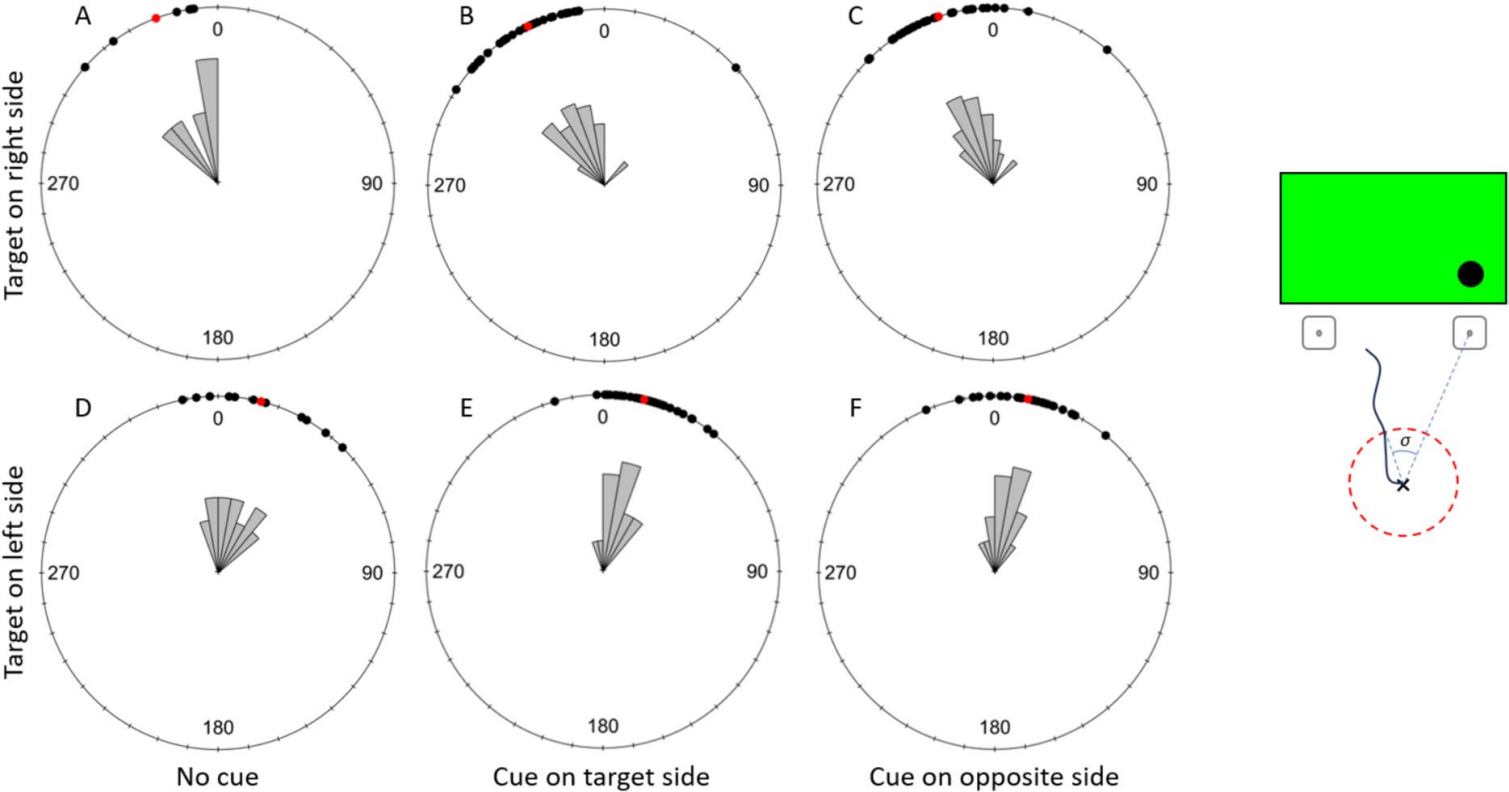
Detection task: effect of the cuing condition on flight direction relative to the target (σ) at 1 cm from the take-off point. The top row shows trials with the target displayed on the right side of the screen and the bottom row shows trials with the target displayed on the left. **A** and **D** represent the uncued condition, **B** and **E** present data for trials with the target side cued and **C** and **F** show trials with the opposite side cued. Black dots represent individual trials, and red dots show the mean direction for each condition. 0 indicates the direction of the target. A clockwise rotation shows deviation towards the right of the target and counterclockwise indicates a deviation to the left of the target. The drawing on the right shows a schematic representation of the angle σ. It represents the two chips placed in front of the screen with the target displayed on the right side. The cross shows the bee’s take-off point, and the black line shows its trajectory. The red circle represents the early flight radius (either 1 or 5 cm in our experiment). The dashed blue lines form the angle between the place where the bee crossed the early flight radius and the direction of the correct chip. This angle therefore represents the bee’s early flight direction relative to the chip below the target

**Fig. 6:**
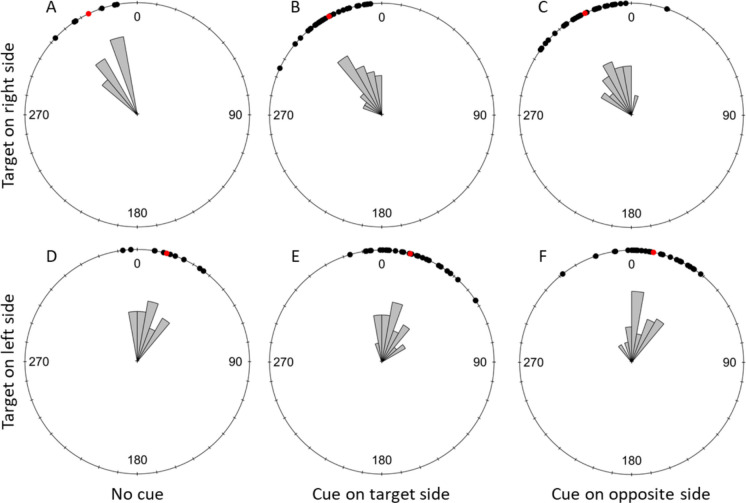
Detection task: Effect of the cuing condition on flight direction relative to the target (σ) at 5 cm from the take-off point. The top row shows trials with the target displayed on the right side of the screen and the bottom row shows trials with the target displayed on the left. A and D represent the uncued condition, B and E present data for trials with the target side cued and C and F show trials with the opposite side cued. Black dots represent individual trials, and red dots show the mean direction for each condition. 0 indicates the direction of the target. A clockwise rotation shows deviation towards the right of the target and counterclockwise indicates a deviation to the left of the target

Bee flights in the first 1 cm from their take-off point were significantly oriented towards the target in all three cuing conditions when the target was on either side of the screen (Fig. 5; Table 2A. The same was true for bee flights 5 cm from their take-off points (Fig. 6; Table 2B).

**Table 2:** Results of the statistical analyses of the bees’ early flight direction during the detection task

A) Early Flight Direction toward the target at 1 cm from take off point	Rayleigh test	
Tested condition	Statistic	p
Uncued with target on left side (11)	0.901	< 0.001
Target side cued with target on left side (*N* = 33)	0.855	< 0.001
Opposite side cued with target on left side (*N* = 30)	0.912	< 0.001
Uncued with target on right side (*N* = 6)	0.923	< 0.001
Target side cued with target on right side (*N* = 33)	0.953	< 0.001
Opposite side cued with target on right side (*N* = 32)	0.960	< 0.001
B) Early Flight Direction toward the target at 5 cm from take off point	Rayleigh test	
Tested condition	Statistic	p
Uncued with target on left side (*N* = 10)	0.877	< 0.001
Target side cued with target on left side (*N* = 34)	0.859	< 0.001
Opposite side cued with target on left side (*N* = 30)	0.876	< 0.001
Uncued with target on right side (*N* = 6)	0.938	< 0.001
Target side cued with target on right side (*N* = 32)	0.922	< 0.001
Opposite side cued with target on right side (*N* = 31)	0.939	< 0.001
C) Early Flight Direction comparison in the three cueing conditions at 1 cm	Watson-Wheeler test	
Target side	W	p
Target on left side (*N* = 74)	5.239	0.264
Target on right side (*N* = 71)	4.817	0.307
D) Early Flight Direction comparison in the three cueing conditions at 5 cm	Watson-Wheeler test	
Target side	W	p
Target on left side (*N* = 74)	0.654	0.957
Target on right side (*N* = 69)	2.188	0.701

The table shows the statistics and *p*-values for the Rayleigh tests analysing whether the bees’ flight direction was significantly oriented towards the target, at 1 cm (A) and 5 cm (B) from their take-off point. It also shows the statistics, degrees of freedom and *p*-values of the Watson-Wheeler tests comparing the bees’ early flight direction in the three cueing conditions (uncued, cue on the target side and cue on the opposite side) when the target was on the left or the right side of the computer screen. N indicates the sample size used for each test

The direction of trajectories at 1 cm from the take-off point did not significantly differ between cuing conditions when the target was presented on the right or the left side of the screen (Table 2C). The same was true at 5 cm from the bees’ take-off point (Table 2D).

Because our exclusion criteria were very conservative, we ran the same analyses for the early flight directions at 1 and 5 cm without excluding any flights and the results obtained were identical.

### Discrimination Task

#### Final Choice

**Fig. 7:**
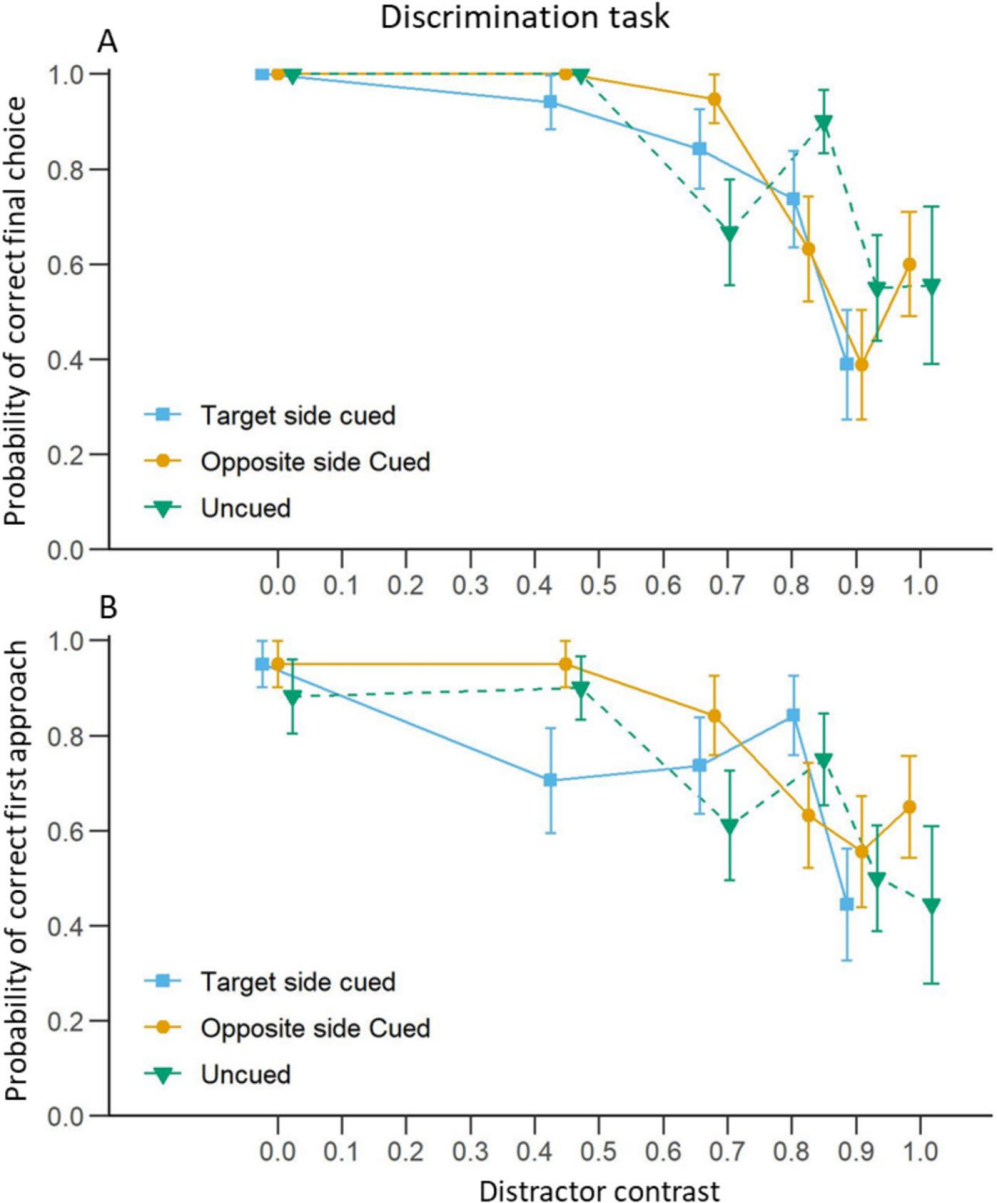
Discrimination task: Effect of distractor contrast and cuing condition on bee choices. **A**) Mean (± S.E.) proportion of trials in which bees chose the target chip as a function of the Michelson contrast of the distractor. **B**) Mean (± S.E.) proportion of trials in which bees first approached the target chip at less than 10 cm. Blue curves represent trials where the cue was presented on the same side as the target. Yellow curves represent trials where the cue was presented on the opposite side to the target. Green curves represent trials without a cue

In the discrimination task, increasing distractor contrast significantly decreased the probability that bees chose the target location in all cuing conditions (Fig. 7A; Table 3A). This reflects the fact that increasing distractor contrast made the target and distractor more difficult to discriminate.

**Table 3:** Results from the Generalised Linear Models and the Generalised Linear Mixed Models used to analyse the final choice and first approach data during the discrimination task

A) Final Choice	GLM	*N* = 313		
Tested effect	Estimate	Std error	Z	p
Distractor contrast (for Uncued)	−5.339	1.737	−3.074	0.002
Distractor contrast (for Target side cued)	−7.518	2.393	−3.142	0.002
Distractor contrast (for Opposite side cued)	−7.493	1.989	−3.768	< 0.001
Monitor side cued (Target side cued VS Uncued)	1.372	2.440	0.562	0.574
Monitor side cued (Opposite side cued VS Uncued)	1.772	2.288	0.774	0.439
Monitor side cued (Opposite side cued VS Target side cued)	0.400	2.628	0.152	0.879
Interaction Distractor contrast : Monitor side cued (Target side cued VS Uncued)	−2.179	2.956	−0.737	0.461
Interaction Distractor contrast : Monitor side cued (Opposite side cued VS Uncued)	−2.154	2.640	−0.816	0.415
Interaction Distractor contrast : Monitor side cued (Opposite side cued VS Target side cued)	0.025	3.111	0.008	0.994
B) First Approach	GLM	*N* = 239		
Tested effect	Estimate	Std error	Z	p
Distractor contrast (for Uncued)	−2.536	0.909	−2.791	0.005
Distractor contrast (for Target side cued)	−2.327	0.955	−2.437	0.015
Distractor contrast (for Opposite side cued)	−3.354	1.090	−3.076	0.002
Monitor side cued (Target side cued VS Uncued)	−0.066	0.992	−0.066	0.947
Monitor side cued (Opposite side cued VS Uncued)	1.064	1.149	0.926	0.355
Monitor side cued (Opposite side cued VS Target side cued)	1.130	1.147	0.985	0.325
Interaction Distractor contrast : Monitor side cued (Target side cued VS Uncued)	0.209	1.318	0.159	0.874
Interaction Distractor contrast : Monitor side cued (Opposite side cued VS Uncued)	−0.818	1.419	−0.576	0.564
Interaction Distractor contrast : Monitor side cued (Opposite side cued VS Target side cued)	−1.027	1.449	−0.709	0.479
C) First Approach Duration (depending on cueing conditions)	GLMM	*N* = 239		
Tested effect	Estimate	Std error	Z	p
Distractor contrast (for Uncued)	−0.206	0.096	−2.152	0.031
Distractor contrast (for Target side cued)	−0.062	0.092	−0.669	0.504
Distractor contrast (for Opposite side cued)	−0.041	0.059	−0.698	0.485
Monitor side cued (Target side cued VS Uncued)	−0.128	0.093	−1.377	0.168
Monitor side cued (Opposite side cued VS Uncued)	−0.157	0.084	−1.867	0.062
Monitor side cued (Opposite side cued VS Target side cued)	−0.029	0.073	−0.393	0.694
Interaction Distractor contrast : Monitor side cued (Target side cued VS Uncued)	0.144	0.133	1.090	0.276
Interaction Distractor contrast : Monitor side cued (Opposite side cued VS Uncued)	0.165	0.114	1.449	0.147
Interaction Distractor contrast : Monitor side cued (Opposite side cued VS Target side cued)	0.020	0.110	0.183	0.855
D) First Approach Duration (depending on first approach correctness)	GLMM	*N* = 239		
Tested effect	Estimate	Std error	Z	p
Distractor contrast (for Correct first approaches)	−0.087	0.048	−1.825	0.068
Distractor contrast (for Incorrect first approaches)	−0.327	0.084	−3.914	< 0.001
First approach correctness (Correct first approaches VS Incorrect first approaches)	0.254	0.073	3.484	< 0.001
Interaction Distractor Contrast : First approach correctness	−0.240	0.098	−2.441	0.015

Details as in Table 1. The table shows the effect of the target contrast, the cueing condition, and their interaction on the probability of correct first choice (A), on the probability of correct first approach (B) and on the duration of the first approach (C). Finally, presents the effect of whether the first approach is to the target (correct first approach) or the distractor (incorrect first approach) on the duration of this first approach

Cuing condition, however, did not influence the final choices of the bees when compared with the uncued condition. When comparing the two conditions where the cue was displayed, the effect of its position (on the side of the target or the side of the distractor) did not differ either.

#### First Approach

Similar results were obtained for the probability of a first approach to the target. The bees were less likely to first approach the target side as the distractor contrast increased in the three cuing conditions (Fig. 7B; Table 3B). Here again, the cuing condition did not influence the probability of a first approach to the target when compared to the uncued condition. First approach probabilities in the two conditions where the cue was displayed did not differ either.

#### First Approach Duration

**Fig. 8:**
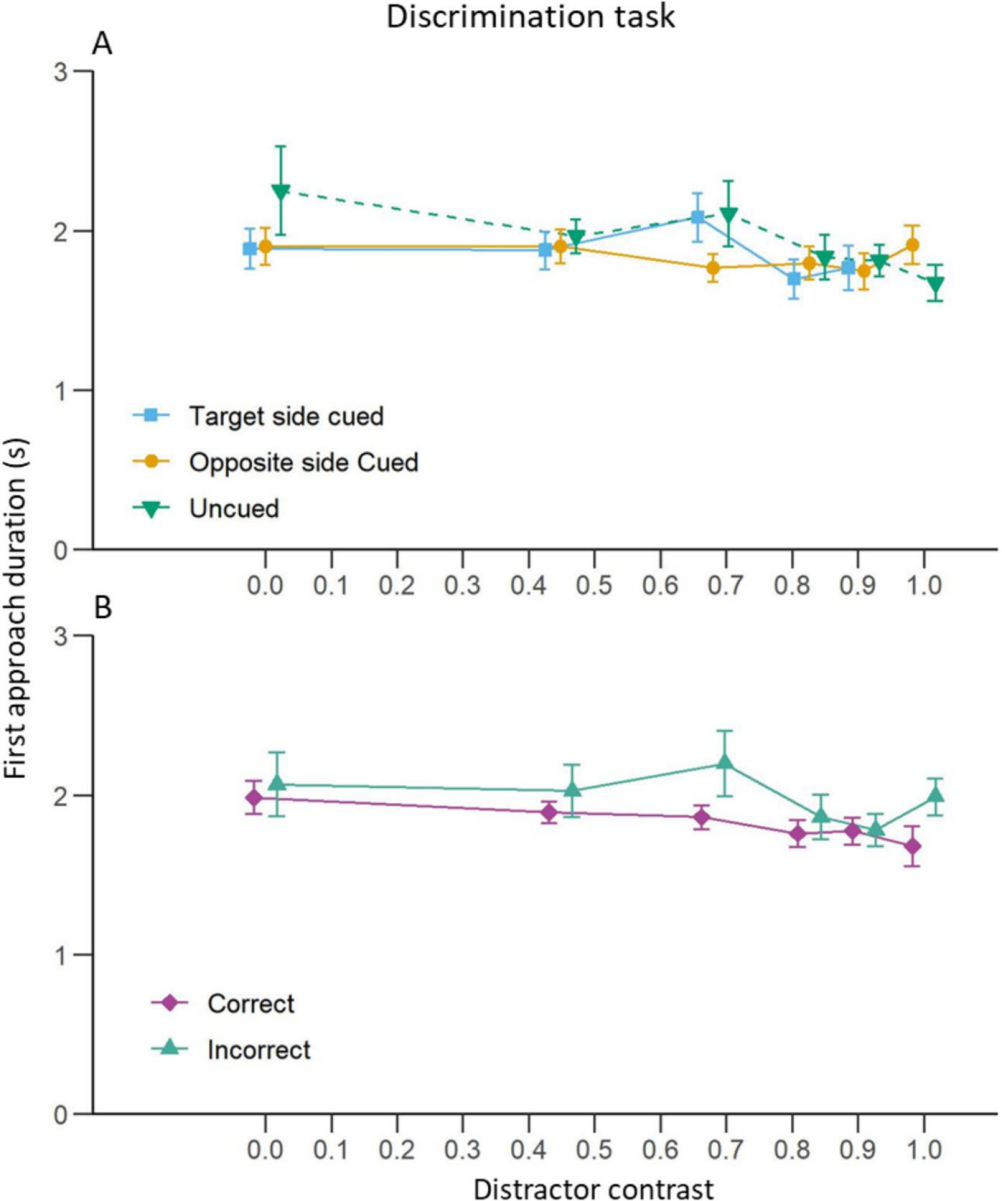
Discrimination task: Effect of distractor contrast on bee first approach duration.** A**) Mean (± S.E.) time taken by the bees to first approach either chip at less than 10 cm for each cuing condition. The blue curve represents trials where the cue was presented on the same side as the target. The yellow curve represents trials where the cue was presented on the opposite side to the target. The green curve represents trials without a cue. **B**) Mean (± S.E.) time taken by the bees to first approach the chip at less than 10 cm for trials where bees made a correct (purple) or incorrect (turquoise) first approach

Surprisingly, bees took less time to approach a chip as the distractor contrast increased in the uncued condition (Fig. 8A; Table 3C). This is probably explained by the fact that, contrary to what we expected, the bees were more willing to approach either chip as the distractor resembled the target more. However, this effect of distractor contrast was not observed in the two cued conditions. Despite this result, the cue did not have a significant effect (main effect or interaction with the distractor contrast) on the first approach duration compared to the uncued condition. Finally, the side of the cue did not have an effect on the duration of the first approach.

Contrary to our results in the detection task experiment, the distractor contrast did not significantly influence the first approach duration in trials where the bee made a correct choice (Fig. 8B; Table 3E) but it did when the bee made a wrong choice for her first approach. This was due to the fact that when bees first approached the distractor’s location, if the distractor was not visible (Michelson contrast = 0), they took more time than when they approached the target. However, an interaction with the contrast indicated that as the distractor became more visible, the bees approached it faster.

#### Early Flight Direction

**Fig. 9:**
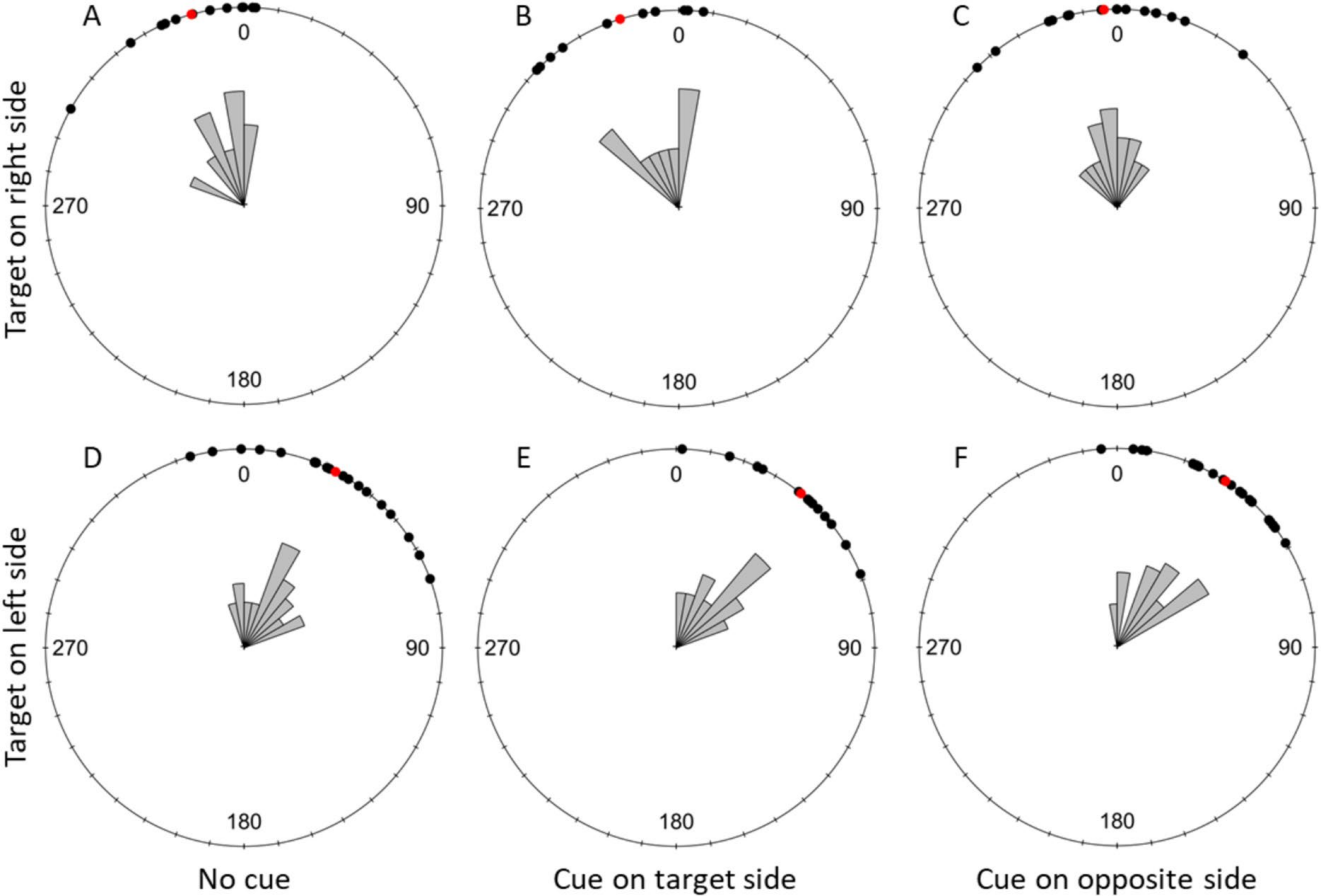
Discrimination Task: Effect of the cuing condition on flight direction relative to the target (σ) at 1 cm from the take-off point. The top row shows trials with the target displayed on the right side of the screen and the bottom row shows trials with the target displayed on the left. **A** and **D** represent the uncued condition, **B** and E present data for trials with the target side cued and **C** and **F** show trials with the distractor side cued. Black dots represent individual trials, and red dots show the mean direction for each condition. 0 indicates the direction of the target. A clockwise rotation shows deviation towards the right of the target and counterclockwise indicates a deviation to the left of the target

**Fig. 10:**
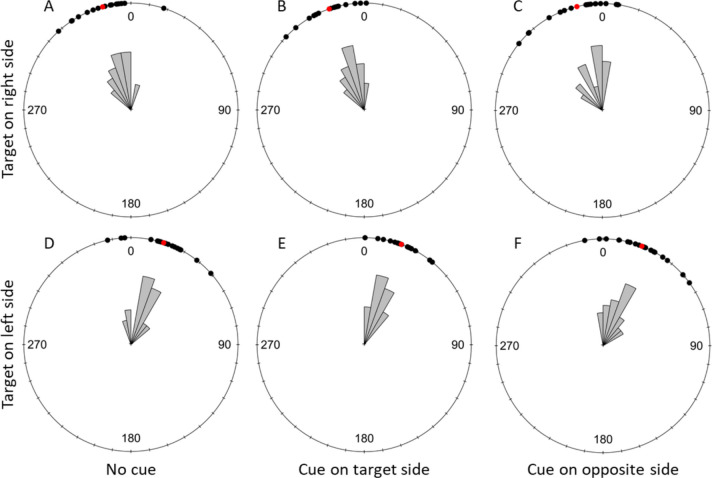
Discrimination task: effect of the cuing condition on flight direction relative to the target (σ) at 5 cm from the take-off point. The top row shows trials with the target displayed on the right side of the screen and the bottom row shows trials with the target displayed on the left. A and D represent the uncued condition, B and E present data for trials with the target side cued and C and F show trials with the distractor side cued. Black dots represent individual trials, and red dots show the mean direction for each condition. 0 indicates the direction of the target. A clockwise rotation shows deviation towards the right of the target and counterclockwise indicates a deviation to the left of the target

At a distance of 1 cm away from their take-off point, bees flew significantly towards the target in every cuing condition both when the target was on the right or the left side of the screen (Fig. 9; Table 4A). The same was observed with the bee flights at 5 cm from their take-off points (Fig. 10; Table 4B).

**Table 4:** Results of the statistical analyses of the bees’ early flight direction during the discrimination task

A) Early Flight Direction toward the target at 1 cm from take off point	Rayleigh test		
Tested condition	Statistic		p
Uncued with target on left side (*N* = 19)	0.821		< 0.001
Target side cued with target on left side (*N* = 13)	0.743		< 0.001
Opposite side cued with target on left side (*N* = 21)	0.798		< 0.001
Uncued with target on right side (*N* = 12)	0.918		< 0.001
Target side cued with target on right side (*N* = 11)	0.899		< 0.001
Opposite side cued with target on right side (*N* = 16)	0.960		< 0.001
B) Early Flight Direction toward the target at 5 cm from take off point	Rayleigh test		
Tested condition	Statistic		p
Uncued with target on left side (*N* = 19)	0.925		< 0.001
Target side cued with target on left side (*N* = 16)	0.925		< 0.001
Opposite side cued with target on left side (*N* = 21)	0.900		< 0.001
Uncued with target on right side (*N* = 17)	0.936		< 0.001
Target side cued with target on right side (*N* = 16)	0.922		< 0.001
Opposite side cued with target on right side (*N* = 19)	0.927		< 0.001
C) Early Flight Direction comparison in the three cueing conditions at 1 cm	Watson-Wheeler test		
Target side	W	df	p
Target on left side (*N* = 53)	3.473	4	0.482
Target on right side (*N* = 39)	2.288	4	0.683
D) Early Flight Direction comparison in the three cueing conditions at 5 cm	Watson-Wheeler test		
Target side	W	df	p
Target on left side (*N* = 56)	2.446	4	0.654
Target on right side (*N* = 52)	5.469	4	0.243

The table presents the statistics and *p*-values for the Rayleigh tests analysing whether the bees’ flight direction was significantly oriented towards the target, at 1 cm (A) and 5 cm (B) from their take-off point. It also presents the statistics, degrees of freedom and *p*-values of the Watson-Wheeler tests comparing the bees’ early flight direction in the three cueing conditions (uncued, cue on the target side and cue on the opposite side) when the target was on the left or the right side of the computer screen. N indicates the sample size used for each test

Finally, early flight direction up to 1 cm from the take off point showed that the distribution of these did not significantly differ across cuing conditions (Table 4C). This was also true for the flight direction at 5 cm from the take off point (Tabe 4D).

We ran the same analyses on the flight directions at 1 and 5 cm without excluding any flights to make sure that the results presented above were not only due to our strict exclusion criteria. The analyses with the full dataset were identical.

## Discussion

Our results show that the main variables influencing the choices of the bees (for both their first approaches and final probing) were the contrast of the target in the detection task and the contrast of the distractor in the discrimination task, similarly to what is observed in vertebrates (Giordano et al. 2009; Ling and Carrasco 2006a; Pestilli et al. 2007; Skottun et al. 1987; Solomon et al. 1997).

### The Effect of Target Contrast

A previous study has shown that bumblebees in a Y-maze test can distinguish a sinusoidal grating of 0.09 cycles per degree (or 11.11°) at a Michelson contrast above 63.6% and a grating of 0.18 cycles per degree (or 5.56°) at contrasts above 81% (Chakravarthi et al. 2016). Although our target and distractor were not gratings and thus might have been easier to perceive for the bees, they subtended a visual angle of around 6.41° from the tunnel entrance (46.92 cm from the target centre). Therefore, we expected that at the lowest contrasts, somewhere between 80 and 60% contrast, the stimuli would not be detectable by the bees from such a distance, and their choice accuracy would start decreasing. This was indeed what we observed in the detection task: the rate of correct choices started decreasing between 0.826 (82.6%) and 0.615 (61.5%) target contrast, with bees more likely to make wrong choices at lower contrasts (see Fig. 4A). Conversely, during the discrimination task, the discrimination between the two targets became more difficult for the bees as the contrast of the distractor increased. There was a particularly strong drop in accuracy with distractor contrast higher than 0.680 (68%), when target and distractor differed by less than 32% (see Fig. 7A).

### First Approach Duration

Target contrast also influenced first approach duration during the detection task, resembling results from detection tasks in human and non-human primates where reducing target contrast increased reaction times (Chen et al. 2008; Harwerth and Levi 1978; Mahadevan et al. 2018; Mihaylova et al. 1999; Musselwhite and Jeffreys 1985; Palmer et al. 2007; Snowden et al. 2001). In our experiment, when the target was barely visible or altogether absent, bees took longer to first approach a chip compared to when the target was clearly visible. This result indicates that as a result of the successful training, bees were really looking for the target and were reluctant to approach a chip when they could not see one, even without a punishment for a wrong choice. One explanation for why bees took longer to approach a chip below a lower contrast target (as seen in Fig. 4B) is that they might have been neophobic to these types of targets. This however seems unlikely as the bees also took a longer time to approach a chip at a location without any target (such as incorrect choices in Fig. 4B) or when there was no target displayed at all on the screen (target contrast = 0 in Fig. 4B). The reluctance to approach a low contrast target most likely thus does not come from a neophobic response but from a struggle to perceive it.

The contrast of the distractor in the discrimination task, however, had the opposite effect on first approach duration. The bees appeared to simply choose one chip (right or wrong) and fly directly towards it. This suggests that our bees did not really compare the target and distractor contrasts but only estimated whether the first detected stimulus was close enough to full contrast and, if so, approached and landed on the chip below it. Such behaviour may reflect a sort of automatic information-processing mechanism (Schneider and Shiffrin 1977, reviewed in Birnboim 2003). It is possible that due to the extended training the bees learnt to automatically detect the first high contrast disk on the screen and approach it. However, this seems unlikely, as automatic information-processing mechanisms generally do not appear in varied tasks (Logan 1988; Schneider and Shiffrin 1977). In our case, we introduced variation in the form of the distractor that could take various contrasts and was associated with a punishment (the taste of quinine). This cost for a wrong choice should prevent the formation of automatic information-processing mechanisms. Nevertheless, to reduce the risks of automatization of the target discrimination process, future attempts to uncover bottom-up attention in bees should also vary the contrast of the target. Alternatively, future studies could decorrelate contrast and reward altogether. For example, following the paradigm used in humans by Liu and colleagues (Liu et al. 2005), bees could be trained to discriminate and choose between two Gabor patches of different orientations, a task we know they are capable to perform (Chakravarthi et al. 2016). The contrast of both stimuli could be varied between tests. One could then examine whether cuing would influence the bees’ ability to discriminate between the two stimuli at various contrasts. Such a protocol would allow for the evaluation of the effect of cuing on bee contrast sensitivity whether they had developed an automatic information-processing mechanism or not.

### Flight Orientation

For the detection task, we expected early flight orientations to be random when the target was invisible in the detection task and to grow more oriented towards the target at higher contrasts. For the discrimination task, we expected bees to be oriented early on towards the target when the distractor was invisible and to get progressively more randomly oriented for higher distractor contrasts. Contrary to these expectations, target contrast did not influence the early flight orientation of bees in the detection task, and the same was true for distractor contrast in the discrimination task. One theoretical explanation for this could be that the bees could not see the target from the entrance of the tunnel. However, our target subtended a visual angle of approximately 6.41° at the entrance and bumblebees can resolve achromatic sinusoidal gratings of 0.21 cycles per degree or 4.76° (Chakravarthi et al. 2016). Other research also shows that bumblebees can detect yellow targets sustaining angles between 3.4° and 7° depending on the size of the bee (Spaethe and Chittka 2003), with only the smallest individuals needing target larger than 6°. Moreover, later studies have demonstrated in Y-maze experiments that bumblebees could detect similar yellow targets (with a green contrast with their background) subtending an angle as small as 2.3° (Dyer et al. 2008) or 1.8° (Wertlen et al. 2008). Thus, it seems unlikely that the bees were unable to resolve the target at the tunnel entrance. It is perhaps more likely that they made their decision at a later point when they were closer to the screen. This would be consistent with the behaviour observed by Guiraud and colleagues (Guiraud et al. 2018) who observed that bees choosing between two complex stimuli in the two branches of a Y-maze did not choose from the entrance of the maze but approached and scanned one stimulus before deciding to attempt to collect the reward or visit the other branch.

More importantly, we did not see any effect of the cue on the bees’ ability to detect the target or discriminate it from the distractor. One possibility is that the bees did not perceive the cue either due to its size or to its duration. However, this seems unlikely. The measured irradiance of the cue against the background provides a strong chromatic and achromatic contrast (achromatic Michelson contrast = 0.83). The cue also subtends an angle of around 8.86° at the tunnel entrance (49.21 cm from the cue centre) which bees would be able to resolve (Chakravarthi et al. 2016; Dyer et al. 2008; Spaethe and Chittka 2003; Wertlen et al. 2008). It is also unlikely that the duration of the cue was too short for the bees to perceive it. The integration time of *Bombus terrestris*’s blue photoreceptors is 9.7 ms (Skorupski and Chittka 2010a), well below the duration of our cue. In addition, this species of bumblebee was behaviourally shown to detect blue stimuli flashed for as short as 25 ms (Nityananda et al. 2014). Thus, the bees should have been able to perceive our cue with a presentation duration of 200 ms. As optimal cue duration for bees is still unknown, we suggest that future work should aim to determine the best cue duration to possibly capture bees’ attention.

### Difference Between Previous Cuing Paradigms for Insects

Flashing cues have been shown capture insect attention. For example, a flashed cue could distract praying mantises away from a simulated prey on a screen (Robert et al. 2025). Interestingly, a stronger effect was also observed in *Drosophila* (Sareen et al. 2011): fruit flies were more likely to follow a vertical bar on a circular screen if it flashed multiple times before moving. Our cuing paradigm does differ from the one used in this experiment. There, the target was identical to the cue and flashed repeatedly at 5 Hz. In our experiment, however, we tried to reproduce cuing paradigms often used with vertebrates (Bowman et al. 1993; Cameron et al. 2002; Fernández et al. 2022; Fernández and Carrasco 2020; Gabay et al. 2013; Guzhang et al. 2021; Hein et al. 2006; Quest et al. 2022; Remington et al. 1992; Wang et al. 2015) and the cue was presented only once (for a duration of 200 ms) rather than flashing on and off. For the same reason, our cue was also distinct from and did not spatially overlap with the target location to avoid a possible masking effect (Breitmeyer and Ogmen 2006).

Similarly to Sareen and colleagues, other experiments have also used cues that are identical to the targets and showed attentional capture in an insect. Lancer et al. (2019) recorded from the CSTMD1 neuron of dragonflies in response to two targets simultaneously moving upward. They showed that the neuron had equal chances to selectively attend to either one of the targets. However, if one target appeared earlier than the second one both temporally and spatially on the screen, it was more likely to elicit a response in the neuron. Here again, the cue and the target were identical, while our paradigm used two distinct visual stimuli for the cue and the target. Finally, as we were trying to observe the effect of attention on a slightly more natural process and to gain insights from their flight trajectories, the bees in our experiment were freely flying compared to the tethered flies and dragonflies in the previous research. All these differences with these previous studies may explain that we could not find any effect of our cue. We were trying to better recreate classic spatial cuing experiments with our paradigm but this appears to not have been effective in capturing attention. If attentional processes in insects are comparable to those of vertebrates, the nature of the cue, its position relative to the target or the time between the cue and target onset would be important to successfully capture the animal’s attention (Franconeri et al. 2005; Franconeri and Simons 2003; Fuller et al. 2009; Lu 2006; Posner and Cohen 1984; Pratt and McAuliffe 2001; Steinman et al. 1997; Tsal 1983). Our results suggest that our cue did not possess the required characteristics or duration to capture bumblebees’ attention.

### The Importance of Different Attentional Processes in Insects

Since worker bumblebees search and forage on rewarding flowers in their environment (Heinrich 1976), it might be more critical for them to evolve a cognitive process resembling vertebrate top-down attention. Such a process would allow them to tune their sensory system to enhance the detection of stimuli they previously associated with the most rewarding flowers in their environment such as their shape or colour (Liu 2019). A previous study has shown that two close insect taxa, honeybees and bumblebees, may process visual information differently during a discrimination task with multiple distractors (Morawetz and Spaethe 2012). In this experiment, honeybees appeared to be more affected by the number of distractors than bumblebees, suggesting that honeybees needed to attend to each object sequentially while bumblebees could rapidly and accurately single out the target(s) among the distractors. This difference might indicate the presence of two different forms of top-down attentional processes in these species. If such close species possess distinct attentional mechanisms, it is conceivable that bottom-up attention has evolved in fruit flies, mantises, or dragonflies (as presented in the paragraphs above) but not in bees.

On the other hand, bumblebees are predated by birds at the entrance of their nest (Goulson et al. 2018) or by wasps (Dukas 2005) and crab spiders while foraging on flowers (Dukas and Morse 2003; Morse 1986; Rodríguez-Gironés and Jiménez 2019). Crab spiders are sit-and-wait predators that are camouflaged on flowers and strike at bumblebees after they land to forage. To avoid being predated by these animals, it would be beneficial to have the ability to prioritise sudden changes in the environment (such as a predator attack). Having a faster reaction time as a result would be essential for the workers’ survival. As the predation on workers decreases bumblebee colony fitness (Goulson et al. 2018), with an especially strong effect of crab spiders’ predation (Cresswell 2017), we should expect some form of bottom-up attention in bumblebees to help them evade attacks. Given our results, investigating this would likely need different approaches to the one we took. For example, to mimic a more ecologically relevant predator avoidance situation, one could train bees to associate a visual stimulus with a punishment — either electrical (Abramson 1986; Mota et al. 2011; Núñez 1997) or mechanical (Ings and Chittka 2008; Nityananda and Chittka 2015; Wang et al. 2013) — when landing on a flower marked by this stimulus, in a paradigm comparable to the one of Nityananda and colleagues (2015). However, the use of a computer screen would allow for the decrease of the contrast of the punished stimulus as well as the brief flashing of the stimulus just before the landing of the bee during test. This rapid flashing could mimic the sudden movement of striking crab spider. One could then observe if the flash increased the perceived contrast of the punished stimulus in the tested bees. Such a protocol would also have the advantage of not having a separate cue but, instead, flashing the target itself, which was a common factor to the two successful bottom-up attention studies in insects (Lancer et al. 2019; Sareen et al. 2011).

Recently, studies have shown that fruit flies have dedicated neural pathways responding to visual looming cues and their characteristics such as size and direction to generate fast and directed non-stereotypical escape behaviours, overriding other behaviours (Ache et al. 2019; Card and Dickinson 2008; de Vries and Clandinin 2012). Such a rapid and spontaneous analysis of a stimulus strongly resembles bottom-up attention. This suggests that looming cues may be better suited to investigate similar attention-like processes in insects. The rapid development of virtual reality and insect flight simulators could help with the investigation of the effect of such stimuli (Eckel et al. 2025; Frasnelli et al. 2018; Geng et al. 2022; Lafon et al. 2021, 2023; Rusch et al. 2017). They could allow for the precise and flexible manipulation of the animals’ visual environment while preserving their behavioural freedom. For example, with such tools, one could model and simulate the looming of a predator towards the bee in a realistic visual environment (Ogawa et al. 2025), systematically varying and testing parameters to determine which are critical to the successful capture the insect attention. These systems open exciting perspectives for the future of insect attention research.

## Supplementary Information

Below is the link to the electronic supplementary material.

Supplementary file1 (PDF 552 KB)

## Data Availability

The full datasets and R codes used for the analyses are freely available at this link: https://doi.org/10.25405/data.ncl.28847825
